# Effects of Electronic Cigarettes on Indoor Air Quality and Health

**DOI:** 10.1146/annurev-publhealth-040119-094043

**Published:** 2020-01-07

**Authors:** Liqiao Li, Yan Lin, Tian Xia, Yifang Zhu

**Affiliations:** 1 Department of Environmental Health Sciences, Jonathan and Karin Fielding School of Public Health, University of California, Los Angeles, California 90095-1772, USA; 2 Department of Medicine, David Geffen School of Medicine, University of California, Los Angeles, California 90095-1772, USA

**Keywords:** particulate matter, PM_2.5_, ultrafine particles, nicotine, respiratory and cardiovascular effects

## Abstract

With the rapid increase in electronic cigarette (e-cig) users worldwide, secondhand exposure to e-cig aerosols has become a serious public health concern. We summarize the evidence on the effects of e-cigs on indoor air quality, chemical compositions of mainstream and secondhand e-cig aerosols, and associated respiratory and cardiovascular effects. The use of e-cigs in indoor environments leads to high levels of fine and ultrafine particles similar to tobacco cigarettes (t-cigs). Concentrations of chemical compounds in e-cig aerosols are generally lower than those in t-cig smoke, but a substantial amount of vaporized propylene glycol, vegetable glycerin, nicotine, and toxic substances, such as aldehydes and heavy metals, has been reported. Exposures to mainstream e-cig aerosols have biologic effects but only limited evidence shows adverse respiratory and cardiovascular effects in humans. Long-term studies are needed to better understand the dosimetry and health effects of exposures to secondhand e-cig aerosols.

## INTRODUCTION

An electronic cigarette (e-cig) is a battery-powered nicotine delivery system widely used as an alternative to tobacco cigarettes (t-cigs). Since 2011, the global e-cig market has grown rapidly and is projected to reach $48.9 billion by 2025, more than 70% of the market being in North America and Europe (2). The number of e-cig users has also increased markedly, especially among adolescents, despite the US Food and Drug Administration (FDA)’s prohibition of sales to persons under the age of 18 (136). In the United States, the total number of current e-cig users in middle schools and high schools increased from 0.3 million in 2011 to 3.6 million in 2018 (23). Similar increases among adolescents have been observed in other countries, such as the United Kingdom, Canada, South Korea, New Zealand, Finland, and Poland (9, 12).

For t-cigs, the combustion of tobacco leaves releases nicotine and substantial amounts of toxic by-products. In comparison, e-cigs deliver nicotine by vaporizing e-liquids, which typically use propylene glycol (PG) and vegetable glycerin (VG) as the suspension media for nicotine and various flavorings. E-cigs are marketed as a safer alternative to t-cigs because they are combustion-free, yet their use has also increased greatly among nonsmokers (88). Because e-cigs are easy to use, have appealing flavors, and are perceived risk-free (12, 20), they are becoming the most popular tobacco product among adolescents. The newly introduced pod-based e-cigs (e.g., JUUL), which use nicotine salts, offer a wide range of flavors, and have a sleek design, are even more attractive to adolescents than are regular tank-based e-cigs (20, 48, 57, 87). The impacts of e-cigs on population health remain largely unknown; however, with increasing evidence on their biological effects from in vitro and in vivo studies (107), the safety of e-cigs has become a serious public health concern.

Not only active users but also bystanders may be exposed to e-cig aerosols. Estimates indicate that more than 70% of inhaled e-cig aerosols are eventually exhaled (143), which may cause secondhand exposures. E-cigs are commonly used in many places, such as homes, cars, restaurants, bars, and workplaces (46), where vulnerable populations, such as children, adolescents, and pregnant women, might be exposed (27, 139). Secondhand exposure in indoor environments is of particular concern because people typically spend more than 80% of their time indoors (64), where emitted pollutants are not diluted as quickly or as extensively as outdoors. Yet, to what extent such secondhand exposures affect human health is unclear. While a large number of health studies on t-cig secondhand smoke exist (55), data from these studies cannot be extrapolated to e-cigs because the emission characteristics are different.

**Figure 1:**
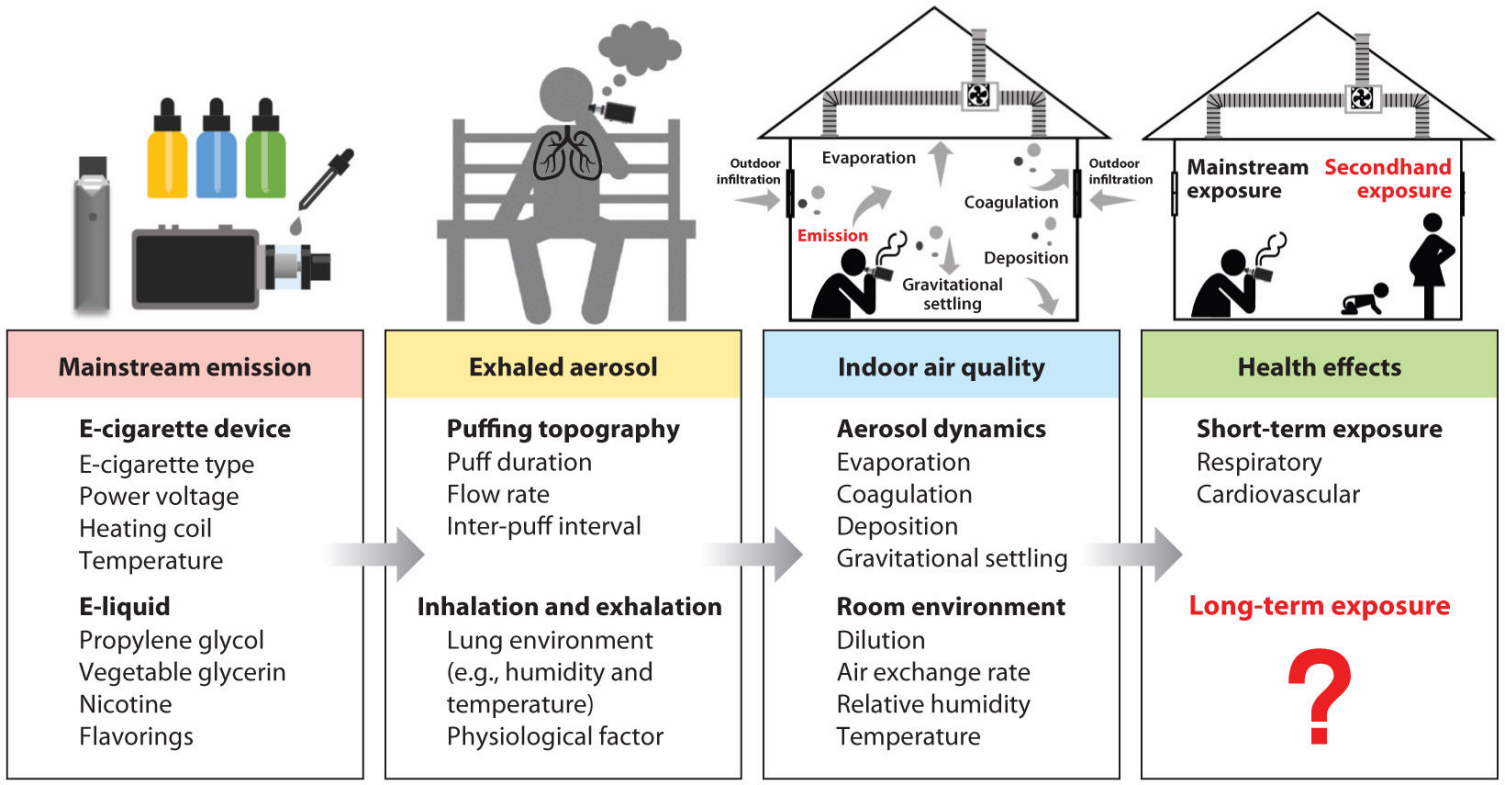
Schematic process from electronic cigarette emissions, to secondhand exposures, and to potential health effects.

To provide a better understanding of the public health risks associated with secondhand exposures to e-cig aerosols, we summarize here the evidence for the impacts of e-cigs on indoor air quality and human health. The schematic process from e-cig emissions, to secondhand exposures, and to potential health effects is summarized in Figure 1.

## THE LITERATURE SEARCH

We reviewed articles published in English and listed on PubMed and the Web of Science before April 2019 that investigated either particulate matter (PM) or chemical compositions in e-cig aerosols, as well as associated health effects. Inclusion criteria include (*a*) e-cig aerosol studies on fine particulate matter (PM_2.5_, particles with aerodynamic diameters ≤2.5 μm) or ultrafine particles (UFPs; particles with a diameter ≤100 nm), (*b*) both mainstream and secondhand exposure studies on chemical compositions in e-cig aerosols, and (*c*) active and passive human exposure studies on e-cig health effects. Key search terms included “electronic cigarette” OR “e-cigarette” OR “e-cig” OR “vaping” in combination with “aerosol” OR “particle” OR “particulate matter (PM),” “chemical composition” OR “chemical emission” OR “exposure,” “mainstream” OR “secondhand” OR “indoor air quality,” and “health.” We extracted data on particle and chemical concentrations from the text, tables, or supporting information in the identified articles. To compare the results across different studies, we report chemical compositions in mass per puff for mainstream and mass per cubic meter (m^3^) for secondhand studies, respectively.

## IMPACTS OF E-CIGS ON INDOOR AIR QUALITY

Studies evaluating the effects of e-cigs on particulate matter (PM) were typically conducted either in a room (>30 m^3^) or in a chamber (<1 m^3^). Studies conducted in rooms represent real-world exposures but were often affected by various environmental factors, such as relative humidity, air exchange rate, and temperature, which are difficult to fully control. On the other hand, chamber studies usually have sufficient control over environmental factors and thus can systematically isolate and investigate the effects of e-cig devices and e-liquid compositions. However, the concentrations of pollutants reported in chamber studies are often orders of magnitude higher than in real-world indoor environments. In the following sections, we focus on studies conducted in real-world indoor environments.

### Indoor Particle Concentrations

**Figure 2:**
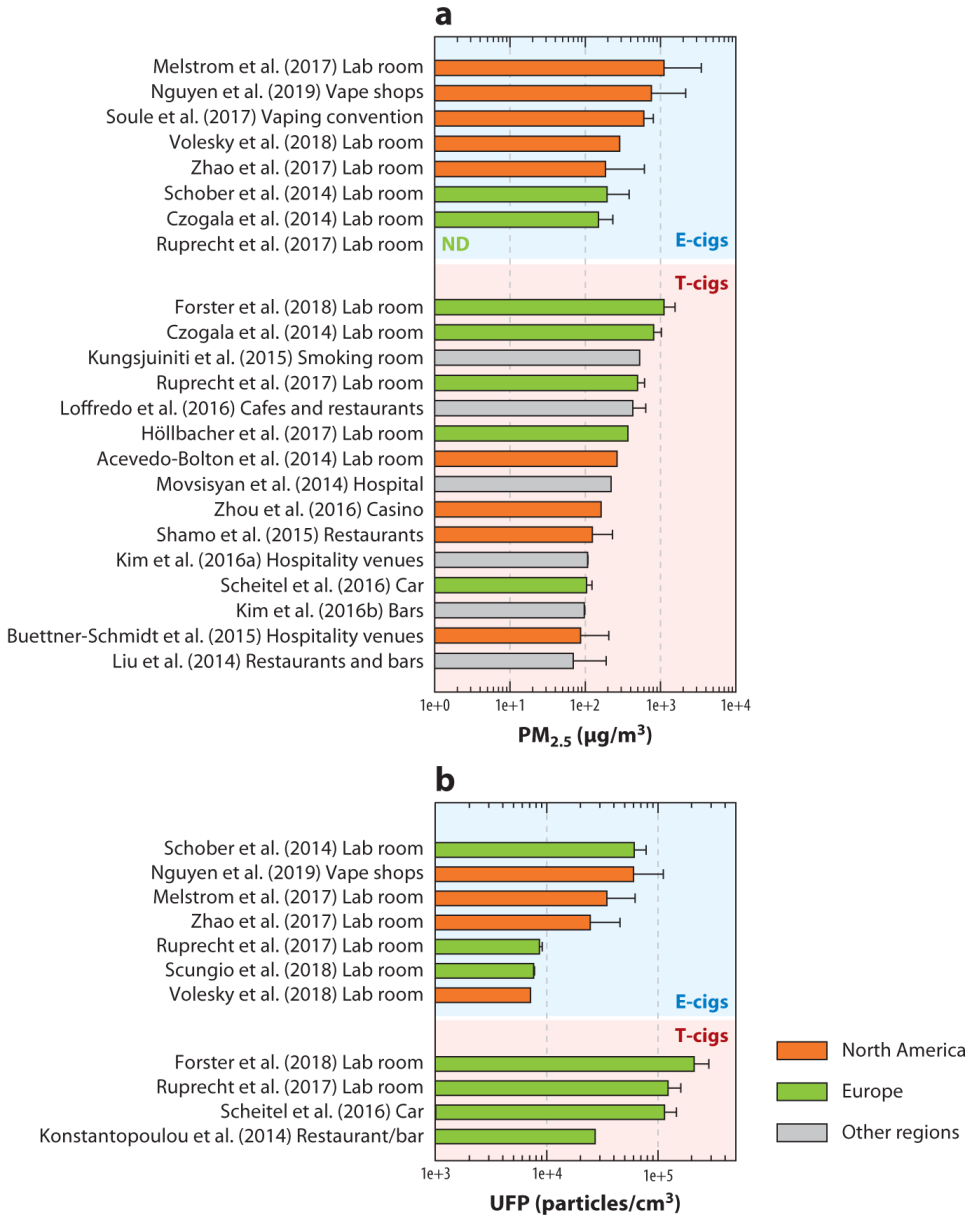
Average concentration of (*a*) PM_2.5_ and (*b*) ultrafine particles (UFPs) in secondhand electronic cigarette (e-cig) aerosols reported for various indoor environments (i.e., laboratory settings and real-world public indoor spaces), by region. Data are from 11 studies on e-cigs and 16 studies on tobacco cigarettes (t-cigs) that reported mean PM_2.5_ and UFP in a laboratory or public indoor environment (1, 13, 24, 40, 56, 70, 71, 74, 77, 82, 83, 89, 96, 97, 113, 117, 118, 120, 121, 125, 138, 146, 149). Abbreviation: ND, not detected.

Exposure to outdoor PM_2.5_ is a well-established risk factor for respiratory and cardiovascular diseases (14). Several indoor studies have reported high concentrations of PM_2.5_ resulting from e-cigs (Figure 2a), which could reach up to 1,121 μg/m^3^, or ~45 times as high as the World Health Organization’s recommended 25 μg/m^3^ limit for 24-h outdoor concentrations (140). In most cases, the reported indoor PM_2.5_ levels during e-cig use are above 150 μg/m^3^, which are similar to those produced by t-cigs. The impacts of e-cigs on indoor air quality are also similar to, if not greater than, other combustion-free nicotine delivery systems, such as waterpipe and “heat-not-burn” products (40, 41, 113). The PM_2.5_ concentrations of 600 to 800 μg/m^3^, as reported in vape shops and vaping conventions (97, 125), are about twice as high as those in hookah bars (148). In comparison, the PM_2.5_ concentrations observed across a wide range of common indoor environments without e-cigs, such as homes, offices, schools, and daycare, are from 8 to 52 μg/m^3^ (95).

In addition to PM_2.5_, UFPs are also of great health concern (42) because they have a greater surface area per unit mass than do larger particles, so they can bind to more toxic chemicals (132). The indoor UFPs during e-cig use can increase up to 20 times over the baseline concentration (7.2 × 10^3^ to 6.2 × 10^4^ particles/cm^3^; Figure 2b) (52) but are still lower than those from t-cigs (7 × 10^4^ to 2.1 × 10^5^ particles/cm^3^) (40, 74, 113, 117) and other combustion-free nicotine delivery systems (7.7 × 10^3^ to 3.2 × 10^5^ particles/cm^3^) (40, 41, 113). Similar to PM_2.5_, indoor UFP concentrations during e-cig use are also higher than those across the wide range of common indoor environments without e-cigs (95).

As shown in Figure 2, studies on t-cig secondhand smoke have been conducted worldwide, but studies on e-cigs are mainly from North America and Europe. The current prevalence of e-cigs in other countries and regions is relatively low, but the e-cig market in many populous countries (e.g., China) is expanding rapidly (109). Thus, secondhand exposures to e-cig aerosols will likely become a potential public health problem in those countries soon. Detailed information on PM_2.5_ and UFP levels, background concentrations, emission protocol, room size, and air exchange rate for both e-cig and t-cig room studies is summarized in Supplemental Table 1.

### Factors Affecting Indoor Particle Concentrations

In addition to particle concentrations, particle size distribution is also important to respiratory health because smaller particles (especially UFPs) generally penetrate deeper into the lung (54). E-cig particles are primarily in the submicron size range (42, 62), exhibiting a bimodal size distribution; one mode is located ~15–30 nm and the other is ~85–100 nm (119, 120, 146). Both particle concentration and particle size distribution are affected by emissions from the e-cig device, exhalation by the e-cig user, and indoor environmental factors, as illustrated in Figure 1 and discussed below.

E-cig emissions are affected by various intrinsic factors, including the e-cig device type, heating coil temperature, power voltage, and e-liquid compositions (7, 39, 42, 45, 68, 105, 122, 123, 147). E-cigs have evolved quickly over time from the cigalike, to the more advanced tank style with customizable voltage and e-liquids, to the recent pod-based vaping systems (47, 57, 87). The power voltage that determines the heating coil temperature has been associated with particle emissions (39, 45). The tank style, which allows a higher voltage, can produce more particles than the cigalike type (89). In addition, particle emissions from e-cigs are also influenced by the e-liquid compositions. The presence of nicotine (42) and higher PG/VG ratios tend to produce more particles (7). The recently introduced pod-based JUUL has not been well studied and raises even more health concerns owing to its high nicotine content in protonated salt and its popularity among adolescents (48, 87).

Variables related to the e-cig user, such as the puffing topography (i.e., flow rate, puff duration, and interpuff interval) and the process of inhalation and exhalation, also affect particle concentrations. In general, the particle concentration increases with higher puffing flow rates, longer puff durations, and shorter interpuff intervals (19, 45, 97, 147). E-cig particles tend to grow in human lungs under high humidity owing to the hygroscopic effect (106, 124). Other physiological factors in the respiratory system, such as lung capacity, air flow, and breath pattern, might also affect e-cig aerosol dynamics. Unfortunately, none of these factors have been studied, and the differences between the inhaled mainstream and the exhaled secondhand e-cig aerosols remain unknown.

Once released into the room air, e-cig particles are subject to aerosol dynamics under certain environmental conditions. In contrast to t-cig smoke, e-cig particles mainly consist of droplets that are more volatile because the e-liquid main ingredient, PG, has a relatively high saturation vapor pressure. E-cig particles have been observed to evaporate within seconds (146). At high particle number concentrations, coagulation is also an important particle-removal mechanism that reduces the number of particles but increases particle size (39, 91). In addition, e-cig particles can be removed from the room air by gravitational settling and surface deposition, leading to potential third-hand exposures, a concern that also warrants future study (51). Increasing dilution or air exchange rate may enhance particle evaporation and reduce particle concentrations and particle sizes (39, 62, 90, 97, 144). Similarly, increasing temperature or decreasing relative humidity may also enhance evaporation and reduce particle size (119, 142). Because e-cig particles are markedly dynamic, their concentrations decay rapidly over distances (>1.5 m) from the source (i.e., e-cig users), especially for PM_2.5_ mass concentration (97, 146). Understanding the dynamics of e-cig particles in an indoor environment is important because it can guide exposure assessment and mitigation strategies.

## CHEMICAL COMPOSITIONS OF E-CIG AEROSOLS

**Figure 3:**
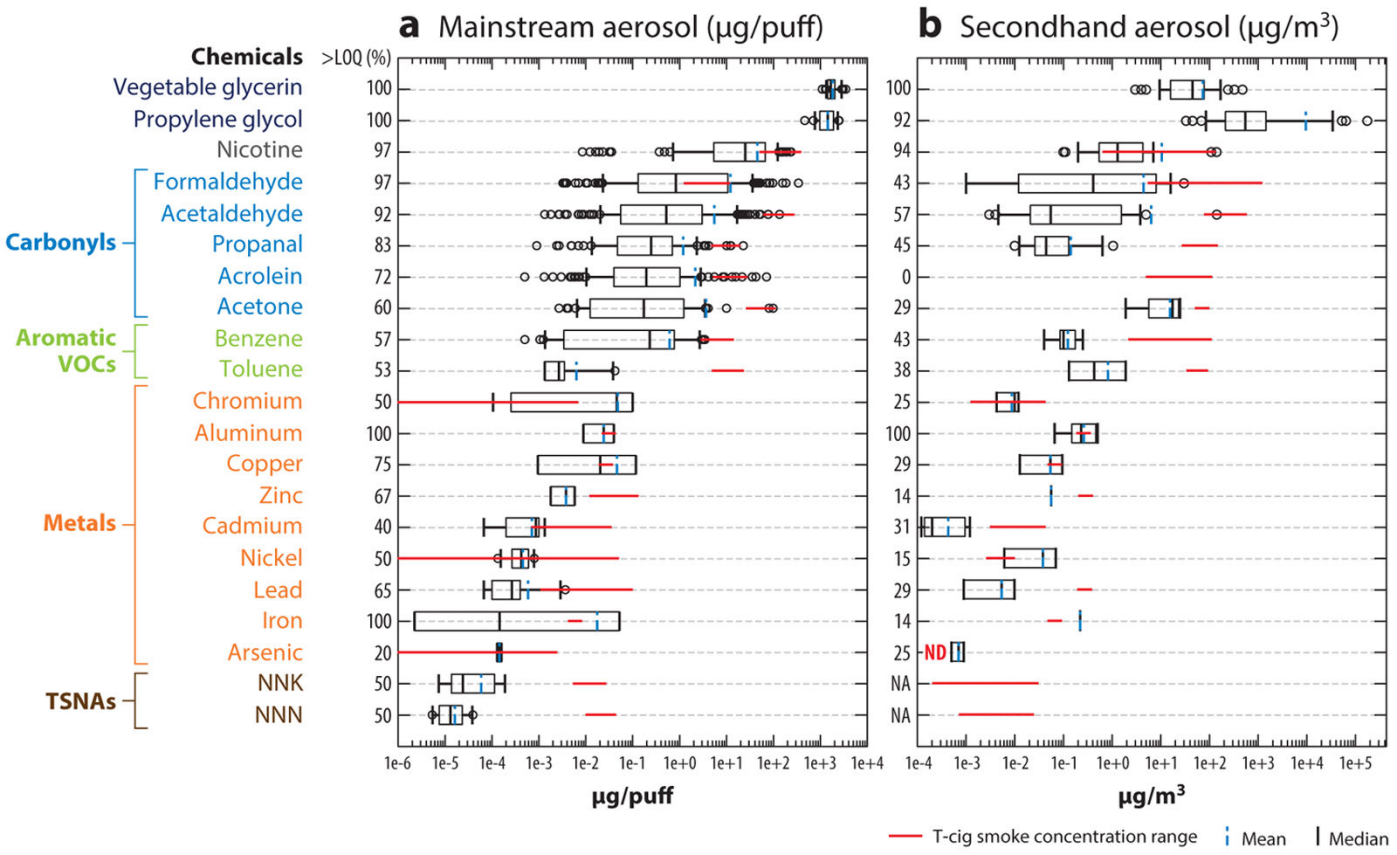
Chemical compositions of (*a*) mainstream electronic cigarette aerosols (μg/puff) from 37 studies and (*b*) secondhand electronic cigarette aerosols (μg/m^3^) from 11 studies. Concentrations of tobacco cig (t-cig)-emitted chemicals are presented as ranges (*red line*) as a reference group. All the data included are background-subtracted values when applicable (8, 18, 21, 24, 25, 28-33, 35, 36, 43, 45, 48-50, 59, 60, 63, 65, 66, 68, 75, 76, 80, 81, 84, 89, 91, 99, 103-105, 111-116, 118, 119, 122, 123, 126-128, 131, 134, 141, 145). Abbreviations: > LOQ%, the percentage of available data points above the limit of quantification (LOQ); ND, not detected; NNK, 4-(methylnitrosamino)-1-(3-pyridyl)-1-butanone; NNN, N′-nitrosonornicotine; TSNAs, tobacco-specific nitrosamines; VOCs, volatile organic compounds.

The effects of e-cig aerosols on health are determined largely by their chemical compositions. The most commonly reported chemicals in both mainstream and secondhand e-cig aerosols are PG, VG, nicotine, carbonyls, aromatic volatile organic compounds (VOCs), trace metals, and tobacco-specific nitrosamines (TSNAs) (Figure 3; Supplemental Table 2). Many chemicals are present in both gas and particulate phases (119). The partition between gas and particulate phases affects the concentration and fate of e-cig-emitted chemicals and warrants future study.

As shown in Figure 3, the chemical profiles of the mainstream and secondhand e-cig aerosols are similar, but as expected, the concentrations of most chemicals in the secondhand aerosols are much lower than in the mainstream. Overall, the most abundant chemicals detected in the e-cig mainstream are PG and VG, followed by nicotine, carbonyls, aromatic VOCs, and trace metals. Most of the chemicals in the mainstream come from the major components of e-liquids: PG, VG, and nicotine. Although the FDA states that ingesting PG and VG in consumer and household products is safe, inhaling vaporized PG and VG at high concentrations may irritate the lungs (69), which is a unique health risk for e-cig aerosols (81, 84). The significant amount of nicotine reported in the e-cig aerosols also poses several health risks. Adolescents are particularly susceptible to nicotine’s addictive effects (135). Existing evidence indicates that never-smoking youth who are exposed to nicotine through e-cigs are more likely to start smoking compared with naïve e-cig users (47). In addition, nicotine contributes to adverse health effects on the cardiocirculatory, respiratory, and gastrointestinal systems (11, 92).

Other observed chemicals such as formaldehyde, acetaldehyde, propanol, acrolein, acetone, and benzene are likely produced either by dehydration of PG/VG or by the reactions between PG and VG at high heating coil temperatures (99, 105, 112, 122). E-cig-related aldehydes might also come from flavoring additives in the e-liquid (68). Aldehydes are cytotoxic and can produce adverse respiratory effects (58). In addition, formaldehyde and acetaldehyde are classified by the International Agency for Research on Cancer (IARC) as carcinogenic to humans (Group 1) and possibly carcinogenic to humans (Group 2B), respectively (58). Among the aromatic VOCs, the IARC lists benzene as a human carcinogen and toluene may be neurotoxic (58).

The likely sources of trace metals, especially those with relatively higher concentrations (i.e., chromium, aluminum, and copper), are the metal-coated wires of the heating coils (114, 141). Inhaling trace metals may irritate the respiratory system and impair respiration (141). Cadmium, lead, chromium, arsenic, and nickel are also classified as human carcinogens (61). Two studies found nicotine-derived nitrosamines in e-cig aerosols, such as N′-nitrosonornicotine (NNN) and 4-(methylnitrosamino)-1-(3-pyridyl)-1-butanone (NNK) (49, 84), which are strong carcinogens that may cause lung and oral cancers (53). Whether these compounds are products of chemical reactions of nicotine or impurities in the e-liquid is not clear (29).

The concentrations of most chemicals in the e-cig mainstream aerosols are lower than those of t-cigs (Figure 3). The only exceptions are PG and VG, which are major components of e-liquid but are not used in t-cigs. Concentrations of trace metals in mainstream aerosols are similar in e-cigs and t-cigs. However, chromium, a carcinogenic and respiratory toxicant, is at higher concentrations in e-cigs, suggesting potential risks from chromium-coated wire in heating coils (141). The concentration of nicotine in e-cig mainstream aerosol is similar to or slightly lower than that in t-cig smoke. Of note, the nicotine content in a single JUUL pod is higher than that in 20 t-cigs and may lead to potential cytotoxicity and more significant addiction effects (102). The concentrations of carbonyls and aromatic VOCs are 10–1,000 times higher in the mainstream emissions of t-cigs than in e-cigs. Because these compounds are highly toxic, the observed lower concentrations indicate that e-cig aerosols are likely less toxic than t-cig smoke (16, 100).

In addition to the chemicals described above, e-cig aerosols contain a wide variety of VOCs at a much lower level, such as acetonitrile, isoprene, ethanol, diacetyl, and acetoin, which likely originate from the flavoring additives (3, 73, 79, 81). Even weaker evidence exists in the literature on the presence of polycyclic aromatic hydrocarbons, crotonaldehyde, acetol, glyoxal, glycidol, and benzaldehyde in e-cig aerosols.

## HEALTH EFFECTS OF E-CIG AEROSOLS

The chemical profiles of mainstream and secondhand e-cig aerosols are similar (Figure 3), suggesting that the results of the studies on active e-cig use and secondhand exposures likely reflect the health effects of similar chemical mixtures at different doses. As previously reviewed, many studies have shown that e-cig aerosols are safer than t-cig smoke (100). However, substantial evidence indicates that e-cig aerosols are not safe to cells in vitro or animals in vivo. Results from in vitro studies have identified the biologic effects on various cell types, including airway epithelium and vascular endothelium (107, 130). Similarly, e-cig aerosols also impair lung functions in animals, with inflammation and immune abnormalities as the likely underlying mechanisms (107), and perturb the cardiovascular system (101, 110). E-cig aerosols also present marked carcinogenicity (78) and neurological toxicity (98) in animals, in addition to the observed respiratory and cardiovascular effects (22, 98). However, it remains controversial whether the dosages used in animal studies are relevant to human exposures and whether the results are consistent across different species.

The respiratory and cardiovascular effects of e-cig aerosols were also examined in human studies; most of these focused on the effects of active e-cig use, with only a few studies on secondhand exposures. As indicated by circulating concentrations of cotinine, doses in e-cig active exposure studies are usually higher than those in secondhand studies (Table 1). The results of these studies suggest likely short-term effects (≤2 h exposure) of e-cig aerosols on preclinical end points (Figure 1).

### Respiratory Effects

Most human studies on the respiratory system examine the effects of short-term (≤1 h) exposure among a small number of healthy subjects (Table 1). Lung function is one of the most commonly studied end points, but the results of different studies are inconsistent. Active e-cig use by healthy t-cig smokers over 5 min slightly but significantly reduced lung function measures [i.e., forced expiratory volume in 1 s (FEV1) and forced expiratory flow (FEF) 25%] in a randomized crossover trial (34). However, similar effects were not observed in two crossover trials (one randomized and one nonrandomized), in which active e-cig use over 5 min or 30 min did not change any lung function measure among healthy t-cig smokers (37, 137). In another two crossover trials on secondhand e-cig aerosols (one randomized and one nonrandomized), neither 30-min nor 1-h exposures altered the lung function measures among healthy nonsmokers (37, 133). In contrast, more consistent results are reported for airway resistance, which was significantly increased after active e-cig use or passive exposure in two clinical trials, as determined by impulse oscillometry (133, 137). In addition, a case-control study found substantially altered respiratory proteomic profiles among e-cig users, indicative of impending airway obstruction (44). Nevertheless, the clinical importance of these early changes is not clear. Also unclear is whether increased airway resistance induced by e-cig exposures will worsen over time and eventually contribute to decreased lung function.

Studies have also assessed the short-term effects (≤2 h) of e-cig aerosols on exhaled nitric oxide, a biomarker of airway inflammation associated with increased risk of asthma and bronchitis (129). Three studies report no effect (6, 34, 37), and four studies show either increased (85, 118) or decreased exhaled nitric oxide concentrations after the exposures (133, 137). In two cross-sectional studies of adolescents, e-cig use was significantly associated with greater odds of asthma attacks [odds ratio (OR) = 1.12; 95% confidence interval (CI) (1.01–1.26)] (72) and chronic bronchitis symptoms [OR = 1.70; 95% CI (1.11–2.59)] (86). Likewise, passive exposures to e-cig aerosols were associated with asthma attacks in cross-sectional studies of adolescents with a history of asthma [OR = 1.27; 95% CI (1.11–1.47)] (10), suggesting potential adverse respiratory effects of secondhand exposures, at least among susceptible populations.

### Cardiovascular Effects

Evidence of secondhand exposures to e-cig aerosols on cardiovascular effects in humans is limited. Nevertheless, the effects of active e-cig use on cardiovascular biomarkers have been frequently documented (Table 1). Both habitual and short-term e-cig use can cause a cardiac-autonomic imbalance, as indicated by heart rate variability. Nicotine has been suggested as a likely cause (93, 94). The existing evidence also suggests that active e-cig use induces systemic oxidative stress and inflammation and impairs endothelial function (6, 15, 17, 94). Although oxidative stress and inflammation are important in the pathogenesis of cardiovascular diseases, to what extent the observed cardiovascular effects of e-cigs are clinically relevant is unclear. The cross-sectional National Health Interview Surveys of 2014 (*n* = 37,000) and 2016 (n = 33,000) found that daily e-cig use was associated with myocardial infarction [OR = 1.79; 95% CI (1.20 to 2.66)] (4), but more evidence is needed, especially from long-term, large-cohort studies, before e-cigs can be causally linked with confidence to cardiovascular diseases.

## CONCLUSIONS AND FUTURE PERSPECTIVES

The evidence in the literature confirms that e-cigs degrade indoor air quality and that bystanders are at risk of secondhand exposure. Indoor particle concentrations attributed to e-cigs are similar to those attributed to t-cigs. Although studies of secondhand e-cig aerosols are limited, their chemical composition profiles are similar to those of mainstream e-cig aerosols but have much lower concentrations. E-cigs generate fewer carcinogenic and toxic compounds than do t-cigs, but they still produce substantial amounts of PG, VG, and nicotine, as well as some toxic compounds such as aldehydes and heavy metals. Current health effect studies in humans focus on the acute effects and early biomarkers. These studies have suggested potential respiratory and cardiovascular effects from e-cig aerosols. However, results from these studies are inconsistent, leading to a call for large-cohort and long-term exposure studies that examine the linkage between e-cigs and clinical end points.

Although the effects of e-cigs on human health are not yet fully understood, the high levels of indoor air pollutants produced by e-cigs call for precautionary measures to protect public health. As of October 2016, 32 countries had banned e-cigs from public spaces (67). In the United States, as of July 1, 2019, 18 states and 861 municipalities have already expanded smoke-free laws to include e-cigs and have prohibited their use in smoke-free places (5). In certain places, such as casinos, bars, and other gaming venues where t-cigs are allowed, e-cig use can even worsen indoor air quality. Until the long-term health effects are fully established, we recommend restricting the use of e-cigs in public indoor spaces to protect bystanders from secondhand exposures.

Given the uncertainties of the chemical products of the heating process and the complexity of flavoring additives, the e-cig design features and e-liquid compositions should be further studied to better understand their effects on e-cig aerosol toxicity as the scientific basis for future regulations. Indoor air pollution due to e-cigs could potentially be reduced by enhancing ventilation and air filtration. Unfortunately, studies on mitigation measures that may inform policy are still limited. Future studies also need to focus on identifying vulnerable populations and monitoring places that may contribute to high levels of secondhand exposures, such as vape shops (102), vaping conventions (125), and other indoor environments with no restrictions on e-cig use.

## Supplementary Material

SI

## Figures and Tables

**Table 1:** Summary of studies on the health effects in humans of active electronic cigarette use and passive exposure to secondhand electronic cigarette aerosols

Study^a^	Design	Study subjects (sample size)	Exposure concentrations	Exposure duration	Health effect assessment	Findings
**Respiratory effects: active e-cig use**						
Flouris et al. 2013 (37)	Randomized crossover trial	Healthy t-cig smokers (*n* = 15)	10.4 puffs, serum cotinine: 60.6 ng/ml	30 min	Lung function, eCO, and eNO	No differences before and after e-cig use
Ferrari et al. 2015 (34)	Randomized crossover trial	Healthy t-cig smokers (*n* = 10) and nonsmokers (*n* = 10)	NA	5 min	Lung function, eCO, and eNO	Reduction in lung function after e-cig use only among smokers
Antoniewicz et al. 2016 (6)	Randomized crossover trial	Healthy seldom smokers (*n* = 16)	10 puffs, plasma cotinine: 4.1 ng/ml	10 min	eNO	No differences before and after e-cig use
Vardavas et al. 2012 (137)	Nonrandomized crossover trial	Healthy t-cig smokers (*n* = 30)	NA		Lung function, eNO, and airway resistance	Increased airway resistance and decreased eNO after e-cig use
Marini et al. 2014 (85)	Nonrandomized crossover trial	Healthy t-cig smokers (*n* = 25)	NA	5 min	eNO	Increased eNO after e-cig use
Schober et al. 2014 (118)	Controlled exposure study	Healthy t-cig smokers (*n* = 9)	132 puffs	2 h	eCO and eNO	Increased eNO after the use of e-cig with nicotine
Dicpinigaitis et al. 2016 (26)	Controlled exposure study	Healthy nonsmokers (*n* = 30)	30 puffs	15 min	Cough reflex sensitivity	Inhibited cough reflex sensitivity after the use of e-cig with nicotine
Ghosh et al. 2018 (44)	Case-control	Healthy nonsmokers (*n* = 18) and e-cig users (*n* = 10)	1.8 puffs/h, serum cotinine: 97.2 ng/ml	NA	Airway proteome	Markedly changed protein profiles in lungs among e-cig users that may have clinical implications for the development of chronic lung diseases
Kim et al. 2017 (72)	Cross-sectional study	Adolescents (ages 12–18 years) (*n* = 216,056)	Self-reported e-cig use in past 30 days: 8% of the total population	NA	Asthma attack in the past 12 months	Higher odds of asthma attack [OR = 1.12; 95% CI (1.01–1.26)] associated with the e-cig use
McConnell et al. 2017 (86)	Cross-sectional study	Adolescents (age ~17 years) (*n* = 2,086)	Self-reported past (24.0%) and current (9.6%) e-cig users	NA	Self-reported chronic bronchitis symptoms and wheeze	Higher odds of chronic bronchitis symptoms [OR = 1.70; 95% CI (1.11–2.59)] associated with past e-cig use
**Respiratory effects: passive exposure**						
Flouris et al. 2013 (37)	Randomized crossover trial	Healthy nonsmokers (*n* = 15)	Serum cotinine: 2.4 ng/ml	1 h	Lung function, eCO, and eNO	No differences before and after the exposures
Tzortzi et al. 2018 (133)	Nonrandomized crossover trial	Healthy nonsmokers (*n* = 40)	120 puffs/h in a 35-m^3^ room	30 min	Lung function, eCO, eNO, and airway resistance	Increased air resistance and decreased eNO after the exposures
Bayly et al. 2019 (10)	Cross-sectional study	Adolescents (ages 11–17 years) with self-reported asthma (*n* = 11,830)	Self-reported exposure in past 30 days: 33% of the total population	NA	Asthma attack in the past 12 months	Higher odds of asthma attack [OR = 1.27; 95% CI (1.11–1.47)] associated with the exposures
**Cardiovascular effects: active e-cig use**						
Flouris et al. 2012 (38)	Randomized crossover trial	Healthy t-cig smokers (*n* = 15)	10.4 puffs, serum cotinine: 60.6 ng/ml	30 min	Complete blood count	No differences before and after e-cig use
Antoniewicz et al. 2016 (6)	Randomized crossover trial	Healthy seldom smokers (*n* = 16)	10 puffs, plasma cotinine: 4.1 ng/ml	10 min	Endothelial function biomarkers	Increased endothelial progenitor cell counts after e-cig use
Poulianiti et al. 2016 (108)	Randomized crossover trial	Healthy t-cig smokers (*n* = 15)	10.4 puffs, serum cotinine: 60.6 ng/ml	30 min	Oxidative stress biomarkers	No differences before and after e-cig use
Moheimani et al. 2017 (93)	Randomized crossover trial	Healthy nonsmokers (*n* = 33)	60 puffs, plasma nicotine: 4.1 ng/ml	30 min	Heart rate variability, blood pressure, and biomarkers of oxidative stress and inflammation	A shift in cardiac autonomic balance after the use of e-cig with nicotine
Carnevale et al. 2016 (15)	Nonrandomized crossover trial	Healthy t-cig smokers (*n* = 20) and nonsmokers (*n* = 20)	9 puffs	NA	Biomarkers of endothelial function and oxidative stress	Changes in biomarkers indicative of increased oxidative stress and decreased endothelia function after e-cig use
Chatterjee et al. 2019 (17)	Controlled exposure study	Healthy nonsmokers (*n* = 10)	16–17 puffs	3 min	Biomarkers of oxidative stress and inflammation	Changes in biomarkers indicative of increased oxidative stress and inflammation after e-cig use
Moheimani et al. 2017 (94)	Case-control study	Healthy nonsmokers (*n* = 23) and e-cig users (*n* = 19)	Plasma cotinine: 3.8–139 ng/ml	1.6 years	Heart rate variability, blood pressure, and biomarkers of oxidative stress and inflammation	A shift in cardiac autonomic balance and an increase in oxidative stress among e-cig users
Alzahrani et al. 2018 (4)	Cross-sectional study	Adults (ages >18 years) (*n* = 69,725)	Self-reported daily e-cig users: 1.1% of the total population	NA	Self-reported history of myocardial infarction	Higher odds of myocardial infarction [OR = 1.79; 95% CI (1.20–2.66)] associated with daily e-cig use
**Cardiovascular effects: passive exposures**						
Flouris et al. 2012 (38)	Randomized crossover trial	Healthy nonsmokers (*n* = 15)	Serum cotinine: 2.4 ng/ml	1 h	Complete blood count	No differences before and after the exposures
Poulianiti et al. 2016 (108)	Randomized crossover trial	Healthy nonsmokers (*n* = 15)	Serum cotinine: 2.4 ng/ml	1 h	Oxidative stress biomarkers	No differences before and after the exposures

Abbreviations: CI, confidence interval; e-cig, electronic cigarette; eCO, exhaled carbon monoxide; eNO, exhaled nitric oxide; NA, not applicable; OR, odds ratio; t-cig, tobacco cigarette.

a Studies with the same design are shown in chronological order based on publication date.
