# Supplementary material for: Effects of Electronic Cigarettes on Indoor Air Quality and Health
Source: Annu Rev Public Health. Author manuscript; Available in PMC 2020 Jul 9. (PMC7346849; doi:10.1146/annurev-publhealth-040119-094043)
Supplement: SI [file NIHMS1606427-supplement-SI.pdf]

Supplemental Material: Annu. Rev. Public Health 2020. 41.
https://doi.org/10.1146/annurev-publhealth-040119-094043

SUPPLEMENTAL MATERIAL

Effects of Electronic Cigarettes on Indoor Air Quality and Health

Liqiao Li[1], Yan Lin[1], Tian Xia[2], and Yifang Zhu[1]*

[1] Department of Environmental Health Sciences, Jonathan and Karin Fielding School of Public Health, University of California, Los Angeles, CA 90095-1772 USA

[2] Department of Medicine, David Geffen School of Medicine, University of California, Los Angeles, CA 90095-1772 USA

Submitted to Annual Review of Public Health for publication

* Corresponding author phone: +1-310-825-4324; fax: +1-310-794-2106; e-mail: yifang@ucla.edu

Supplemental Table 1. Summary of $PM_{2.5}$ and UFP levels, background concentration, emission protocol, room size, and air exchange rate for studies in Figure 2

a. $PM_{2.5}$

Study	Location	Mean, μg/m^3	Background, μg/m^3	Emission Protocol	Room Size, m^3	Air Exchange Rate, h^{-1}
**E-Cig Aerosols**						
Melstrom et al. (2017)	Laboratory room	1121 ± 2430	18 ± 15	3 volunteers use e-cigs for 2 h	53	5.0
Nguyen et al. (2019)	6 vape shops	766 ± 1442	12 ± 6	An average of 3.9 puffs per min generated by e-cig users	168-323	0.1-4.8
Soule et al. (2017)	An e-cig vaping event in a hotel	607 ± 212	3 ± 1	A 2-day e-cig event with 59-86 active e-cig users	4023	NA
Volesky et al. (2018)	Laboratory room	290	2	1 e-cig user generated 7 puffs with 4-s puffing duration	38	NA
Zhao et al. (2017)	Laboratory room	188 ± 433	8 ± 1	1 volunteer used e-cigs with 3-s puffing duration, 3-s hold, and 24s pause for 10 min	80	4.1
Schober et al. (2013)	Laboratory room	197 ± 193	6	3 volunteers used e-cigs for 2 h	45	0.6
Czogala et al. (2014)	Laboratory room	152 ± 87	32 ± 30	5 dual volunteers used e-cigs twice for 5 min with a 30-min interval	39	2.6
Ruprecht et al. (2017)	Laboratory room	ND	0	Volunteers used e-cigs for 2 to 3 h; one puff per min for about 7 min, followed by 3 min hold	48	1.5
**T-Cig Aerosols**						
Forster et al. (2018)	Laboratory room	1129 ± 466	6 ± 4	Volunteers smoked a puff every 30 s for 8 puffs.	38	1.2-7.7

Czogala et al. (2014)	Laboratory room	819 ± 229	32 ± 30	5 volunteers smoked twice for 5 min with a 30-min interval	39	2.6
Kungsjulniti et al. (2015)	Smoking rooms in international airports	533	14	NA	NA	NA
Ruprecht et al. (2017)	Laboratory room	500 ± 128	0	Volunteers smoked for about 7 min with 3-min intervals	48	1.5
Loffredo et al. (2016)	15 cafes and 5 restaurants allowed smoking	433 ± 217	72-81	T-cigs and waterpipes smoking observed at all sampling locations	128-235	NA
Höllbacher et al. (2017)	Laboratory room	374	0	1 volunteer smoked t-cigs for 30 min	30	0.5
Acevedo-Bolton et al. (2014)	Laboratory room	267	3-8	1 volunteer smoked 1 or 2 t-cigs for 5-10 min	121 or 460	NA
Movsisyan et al. (2014)	A hospital before smoking ban	222	NA	High frequency of smoking t-cigs observed in multiple locations in the hospital before smoking ban	NA	NA
Zhou et al. (2016)	A smoking-permitted area (i.e., employee break room) in a casino	164	23	25-43 active smokers in the smoking-permitted area in the casino	NA	NA
Shamo et al. (2015)	78 restaurants before smoking ban	126 ± 109	12 ± 23	20% smoking prevalence, 2.6 active smokers per restaurant, 1.11 burning t-cigs per 100 m^3	NA	NA
Kim et al. (2016a)	62 hospitality venues (i.e., restaurants and bars) before smoking ban	109 ± 2	46 ± 2	Smoking observed in 25 hospitality venues	90-1680	NA

Scheitel et al. (2016)	Smoking in a car	105 ± 21	2	1 volunteer smoked 1 or 2 t-cigs in a car	NA	NA
Kim et al. (2016b)	75 bars before smoking ban	98 ± 2	25 ± 2	Smoking was observed in 68-96% of the 75 sampled bars before smoking ban	NA	NA
Buettner-Schmidt et al. (2015)	135 hospitality venues (i.e., bars and restaurants)	88 ± 122	9	0.3 buring t-cigs per 100 m^3, smoking observed in 42% of the hospitality venues	494 ± 601	NA
Liu et al. (2014)	114 restaurants and bars	70 ± 124	NA	3.7 % smoking prevalence, 0.3 active smokers observed per 100 m^3	NA	4.5 (range: 0-14)

b. UFPs

Study	Location	Mean, particles/cm^3	Background, particles/cm^3	Emission Protocol	Room Size, m^3	Air Exchange Rate, h^{-1}
**E-Cig Aerosols**						
Schober et al. (2014)	Laboratory room	61682 ± 17524	4466	3 volunteers used e-cigs for 2 h	45	0.6
Nguyen et al. (2019)	6 vape shops	60667 ± 52296	14580 ± 7845	an average of 3.9 puffs per min generated by e-cig users	168-323	0.1-4.8
Melstrom et al. (2017)	Laboratory room	34961 ± 27992	3707 ± 823	3 volunteers used e-cigs for 2 h	53	5.0
Zhao et al. (2017)	Laboratory room	24800 ± 21400	6390 ± 158	1 volunteer used e-cigs with 3-s puffing duration, 3-s hold, and 24s pause for 10 min	80	4.1
Ruprecht et al. (2017)	Laboratory room	8657 ± 560	0	Volunteers used e-cigs for 2 to 3 h; one puff per min for about 7 min, followed by 3 min hold	48	1.5

Scungio et al. (2018)	Laboratory room	7690 ± 169	NA	1 volunteer used e-cigs for 10 min at 0.7-1.5 puffs/min	40	0.2
Volesky et al. (2018)	Laboratory room	7169	1389	1 e-cig user generated 7 puffs with 4-s puffing duration	38	NA
**T-Cig Aerosols**						
Forster et al. (2018)	Laboratory room	210000 ± 75500	4297 ± 4639	Volunteers smoked a puff every 30 s for 8 puffs.	38	1.2-7.7
Ruprecht et al. (2017)	Laboratory room	122672 ± 37327	0	Volunteers smoked for about 7 min with 3-min intervals	48	1.5
Scheitel et al. (2016)	Smoking in a car	113998 ± 34382	15545	1 volunteer smoked 1 or 2 t-cigs in a car	NA	NA
Konstantopoulou et al. (2014)	A bar/restaurant before smoking ban	27172	12509	An average of 4 smokers observed	NA	NA

Supplemental Table 2. List of reviewed chemical pollutants for both mainstream and secondhand aerosols and corresponding references

Chemicals	References	
	**Mainstream Aerosols**	**Secondhand Aerosols**
Vegetable Glycerin	Dai et al. (2017); Geiss et al. (2015); Margham et al. (2016); Zhao et al. (2018)	Geiss et al. (2015); Liu et al. (2017); Schober et al. (2014); Schripp et al. (2013)
Propylene Glycol	Dai et al. (2017); Geiss et al. (2015); Margham et al. (2016); Zhao et al. (2018)	Geiss et al. (2015); Johnson et al. (2018); Liu et al. (2017); Schober et al. (2014); Schripp et al. (2013)
Nicotine	Czogala et al. (2014); Dai et al. (2017); El-Hellani et al. (2018); Farsalinos et al. (2018b); Geiss et al. (2015); Goniewicz et al. (2013); Goniewicz et al. (2018); Margham et al. (2016); Pagano et al. (2016); Sleiman et al. (2016); Son et al. (2018); Talih et al. (2016); Tayyarah et al. (2014); Zhao et al. (2018)	Ballbè et al. (2014); Chen et al. (2017); Czogala et al. (2014); Geiss et al. (2015); Johnson et al. (2018); Liu et al. (2017); Melstrom et al. (2017); Schober et al. (2014); Schripp et al. (2013)
Formaldehyde	Farsalinos et al. (2017); Farsalinos et al. (2018a); Farsalinos et al. (2018c); Flora et al. (2016); Flora et al. (2017); Geiss et al. (2015); Gillman et al. (2016); Goniewicz et al. (2014); Jensen et al. (2015); Khlystov et al. (2016); Kosmider et al. (2014); Kosmider et al. (2018); Margham et al. (2016); Ogunwale et al. (2017); Qu et al. (2018); Qu et al. (2019); Salamanca et al. (2018); Samburova et al. (2018); Sleiman et al. (2016); Talih et al. (2016); Talih et al. (2017); Tayyarah et al. (2014)	Johnson et al. (2018); Liu et al. (2017); Ruprecht et al. (2017); Schober et al. (2014); Schripp et al. (2013)
Acetaldehyde	Farsalinos et al. (2018a); Farsalinos et al. (2018c); Flora et al. (2016); Flora et al. (2017); Geiss et al. (2015); Gillman et al. (2016); Goniewicz et al. (2014); Khlystov et al. (2016); Kosmider et al. (2014); Kosmider et al. (2018); Margham et al. (2016); Ogunwale et al. (2017); Qu et al. (2018); Qu et al. (2019); Samburova et al. (2018); Sleiman et al. (2016); Talih et al. (2016); Talih et al. (2017); Tayyarah et al. (2014)	Johnson et al. (2018); Liu et al. (2017); Ruprecht et al. (2017); Schober et al. (2014); Schripp et al. (2013)

Propanal	Farsalinos et al. (2018a); Farsalinos et al. (2018c); Geiss et al. (2015); Goniewicz et al. (2014); Khlystov et al. (2016); Kosmider et al. (2014); Kosmider et al. (2018); Margham et al. (2016); Ogunwale et al. (2017); Qu et al. (2018); Qu et al. (2019); Samburova et al. (2018); Sleiman et al. (2016); Talih et al. (2016); Talih et al. (2017); Tayyarah et al. (2014)	Liu et al. (2017); Schober et al. (2014); Schripp et al. (2013)
Acrolein	Farsalinos et al. (2018a); Farsalinos et al. (2018c); Flora et al. (2016); Flora et al. (2017); Geiss et al. (2015); Gillman et al. (2016); Goniewicz et al. (2014); Khlystov et al. (2016); Kosmider et al. (2014); Kosmider et al. (2018); Margham et al. (2016); Ogunwale et al. (2017); Qu et al. (2018); Qu et al. (2019); Samburova et al. (2018); Sleiman et al. (2016); Talih et al. (2016); Talih et al. (2017); Tayyarah et al. (2014)	Johnson et al. (2018); Liu et al. (2017); Ruprecht et al. (2017); Schober et al. (2014)
Acetone	Farsalinos et al. (2018a); Geiss et al. (2015); Goniewicz et al. (2014); Khlystov et al. (2016); Kosmider et al. (2014); Kosmider et al. (2018); Margham et al. (2016); Ogunwale et al. (2017); Qu et al. (2018); Qu et al. (2019); Samburova et al. (2018); Sleiman et al. (2016); Talih et al. (2016); Talih et al. (2017);	Liu et al. (2017); Schober et al. (2014); Schripp et al. (2013)
Benzene	Flora et al. (2016); Margham et al. (2016); Pankow et al. (2017); Sleiman et al. (2016); Tayyarah et al. (2014)	Liu et al. (2017); Schober et al. (2014); Schripp et al. (2013)
Toluene	Flora et al. (2016); Goniewicz et al. (2014); Margham et al. (2016); Tayyarah et al. (2014)	Liu et al. (2017); Schober et al. (2014); Schripp et al. (2013)
Chromium	Margham et al. (2016); Tayyarah et al. (2014); Williams et al. (2013)	Liu et al. (2017); Saffari et al. (2014); Schober et al. (2014)
Aluminum	Palazzolo et al. (2017); Williams et al. (2013)	Saffari et al. (2014); Schober et al. (2014)
Copper	Lerner et al. (2015); Margham et al. (2016); Palazzolo et al. (2017); Williams et al. (2013)	Saffari et al. (2014); Schober et al. (2014)
Zinc	Margham et al. (2016); Palazzolo et al. (2017); Williams et al. (2013)	Saffari et al. (2014); Schober et al. (2014)
Cadmium	Flora et al. (2016); Goniewicz et al. (2014); Margham et al. (2016); Palazzolo et al. (2017); Tayyarah et al. (2014)	Liu et al. (2017); Saffari et al. (2014); Schober et al. (2014)

Nickel	Goniewicz et al. (2014); Margham et al. (2016); Mikheev et al. (2016); Palazzolo et al. (2017); Tayyarah et al. (2014); Williams et al. (2013)	Liu et al. (2017); Saffari et al. (2014); Schober et al. (2014)
Lead	Goniewicz et al. (2014); Margham et al. (2016); Palazzolo et al. (2017); Tayyarah et al. (2014); Williams et al. (2013)	Saffari et al. (2014); Schober et al. (2014)
Iron	Margham et al. (2016); Palazzolo et al. (2017); Williams et al. (2013)	Saffari et al. (2014); Schober et al. (2014)
Arsenic	Flora et al. (2016); Margham et al. (2016); Mikheev et al. (2016); Palazzolo et al. (2017); Tayyarah et al. (2014)	Liu et al. (2017); Schober et al. (2014)
N'-Nitrosonornicotine (NNN)	Farsalinos et al. (2015); Flora et al. (2016); Goniewicz et al. (2014); Margham et al. (2016); Tayyarah et al. (2014)	NA
4-(methylnitrosamino)-1-(3-pyridyl)-1-butanone (NNK)	Farsalinos et al. (2015); Flora et al. (2016); Goniewicz et al. (2014); Margham et al. (2016); Tayyarah et al. (2014)	NA
